# Polymorphism of *Saccharomyces cerevisiae* Strains in DNA Metabolism Genes

**DOI:** 10.3390/ijms24097795

**Published:** 2023-04-25

**Authors:** Anna S. Zhuk, Artem G. Lada, Youri I. Pavlov

**Affiliations:** 1 Institute of Applied Computer Science, ITMO University, 191002 St. Petersburg, Russia; ania.zhuk@gmail.com; 2 Vavilov Institute of General Genetics, St. Petersburg Branch, Russian Academy of Sciences, 199034 St. Petersburg, Russia; 3 Laboratory of Amyloid Biology, St. Petersburg State University, 199034 St. Petersburg, Russia; 4 Department of Microbiology and Molecular Genetics, University of California, Davis, CA 95616, USA; alada@ucdavis.edu; 5 Eppley Institute for Research in Cancer, Fred and Pamela Buffett Cancer Center, University of Nebraska Medical Center, Omaha, NE 68198, USA; 6 Departments of Biochemistry and Molecular Biology, Microbiology and Pathology, Genetics Cell Biology and Anatomy, University of Nebraska Medical Center, Omaha, NE 68198, USA

**Keywords:** yeast *Saccharomyces cerevisiae*, DNA replication, DNA repair, DNA recombination, genome sequence, genetic polymorphism

## Abstract

Baker’s yeast, *S. cerevisiae*, is an excellent model organism exploited for molecular genetic studies of the mechanisms of genome stability in eukaryotes. Genetic peculiarities of commonly used yeast strains impact the processes of DNA replication, repair, and recombination (RRR). We compared the genomic DNA sequence variation of the five strains that are intensively used for RRR studies. We used yeast next-generation sequencing data to detect the extent and significance of variation in 183 RRR genes. We present a detailed analysis of the differences that were found even in closely related strains. Polymorphisms of common yeast strains should be considered when interpreting the outcomes of genome stability studies, especially in cases of discrepancies between laboratories describing the same phenomena.

## 1. Introduction

Molecular biology and genetic studies using a simple eukaryote, *Saccharomyces cerevisiae* yeast, had, have, and will immensely impact the DNA replication, repair, and recombination (RRR) field [1,2]. Yeast strains that were collected and stored by Emil Hansen in the 1880s were introduced in laboratory practice by Öjvind Winge 50 years later. These strains diverted to several groups of modern yeast strains [3,4]. The strains have similar genetic content but multiple single nucleotide changes, different Ty1 element distributions, and structural variations [5,6]. The evidence has accumulated that some variations affect DNA maintenance genes that might be critical for the performance of this machinery. One of the most known classic examples is the finding that a widely used control strain, W303, carries a mutated *RAD5* gene, allele *rad5-535* [7]. Rad5 is a multifunctional helicase/ubiquitin ligase. The mutation alters the conserved nucleotide binding motif ^535^GXGKT to ^535^RXGKT (changed amino acid is underlined). Strains with the *rad5-535* allele are slightly sensitive to a mutagen MMS and show altered genetic interactions with *soh2* mutants affecting the RNA polymerase mediator complex. Rad5 is implicated in template switch during replication [8], and in translesion DNA synthesis (TLS) [9,10]. Polymorphism in *RAD5* might be a weighty modifier of these processes. Strain yJF1, whose derivatives are used to produce proteins for reconstitutions of replication fork in vitro, is the relative of W303 and possesses this allele [11]. Variation between the strains is a common reason for different rates of replication origin activation [12]. The yeast genome results from ancestral genome duplication [13], and many genes have paralogs that are under less stringent selection and evolve rapidly. Examples are RNR genes that have remained paralogs at various stages of diversification. As a result, some strains with deletion of the gene encoding a major subunit of RNR1 are inviable, e.g., W303, but some are viable, BY4741/S288C [14,15]. Another example is a polymorphism of strains explaining critical parameters connecting telomere length and longevity [16].

Before the next-generation sequencing (NGS) era, discrepancies in the results of DNA repair studies were attributed to unknown variations in the genetic background of the used strains. In one extreme case, it was proposed that unknown genetic differences may affect the interpretation of genetic experiments defining the arrangement of DNA polymerases at the fork [17,18]. Many classic and new yeast strains that are used for studies of DNA metabolism and genome stability have now been sequenced, and the genetic causes of phenotypic differences have been uncovered [19,20]. Here, we analyzed the differences in significant RRR genes among several common laboratory strains and LAN series that we have used for the studies of peculiarities of mutagenesis in diploids by a base analog and APOBEC deaminases with an emphasis on kataegis and the hypermutable fraction of yeast cells [21,22,23]. LAN series are close derivatives of the CG379 strain, which has been used for pioneer studies of the consequences of defective DNA polymerase proofreading, defects of mismatch repair (MMR), or their combination on mutation rates [24,25,26,27,28,29]. A variant of the CG379 strain called E134 [29,30] is one of the primary strains for modeling mutator phenotypes of cancer-associated mutations [31,32]. 

## 2. Results

### 2.1. Characteristics of Strains Examined

**Figure 1:**
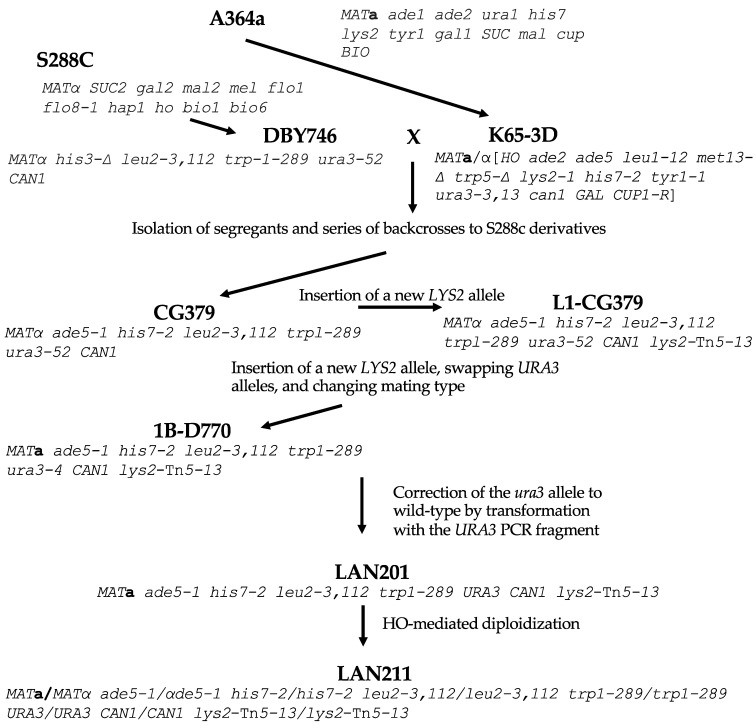
**The genealogy of LAN201 and LAN210 strains.** The principal ancestors are A464a [43] and its derivative K65-3D (brackets in genotype are used to indicate the homozygous diploid genotype of homothallic *HO* strain) [44] on one side, and S288c [4] and its derivative DBY746, on the other [45]. Dr. Craig Giroux (NIEHS, USA) performed a series of backcrosses to find segregants with the desired markers. This yielded CG379, the basic strain for creating DNA polymerase mutations [24]. The *lys2* allele with Tn5 insertion was introduced as described in [46], *ura3-52* was replaced by the *ura3-4* allele, and the strain was made diploid by HO endonuclease. Tetrad dissection gave segregant 1B-D770 with a changed mating type [30]. Next, *ura3-4* was converted back to *URA3* to create LAN201 [22], which gave rise to diploid LAN211 by auto-diploidization with the assistance of an HO-bearing plasmid.

For our comparison, we analyzed the genome of the strain that is the closest relative of the first sequenced yeast strain, S288C, BY4742 (*MATα his3-*Δ*1 leu2-*Δ*0 lys2-*Δ*0 ura3-*Δ*0*). BY4742, and its sibling of a different mating type, BY4741, are widely used for various genetic studies, including genome instability analysis because of the availability of the library of systematic deletions [33,34]. Its genealogy is described in [35]. W303-1A and -1B are *MAT***a** and *MATα* haploids correspondingly, with the same genotype (*ade2-1 ura3-1 his3-11*, *15 trp1-1 leu2-3*, *112 can1-100*) but differ in mating type, originating from W303 diploid parent [36]. W303 is likely closely related to S288C although its history is not well-defined [37]. Less related is SK1, a homothallic strain (*MAT***a***/MATα* [*HO can1 gal2 cup*] [38,39] that is widely used for studies of yeast metabolism and meiosis [40,41]. It is quite distant from S288C [42]. The known parts of the ancestry of LAN201 and LAN211 are summarized in Figure 1. They are derived from CG379 by a series of integration–excisions, one-step gene replacements, mating type switching by HO-containing plasmid, and crosses.

### 2.2. DNA Sequence Variations in Genomes of the Yeast Strains

**Figure 2:**
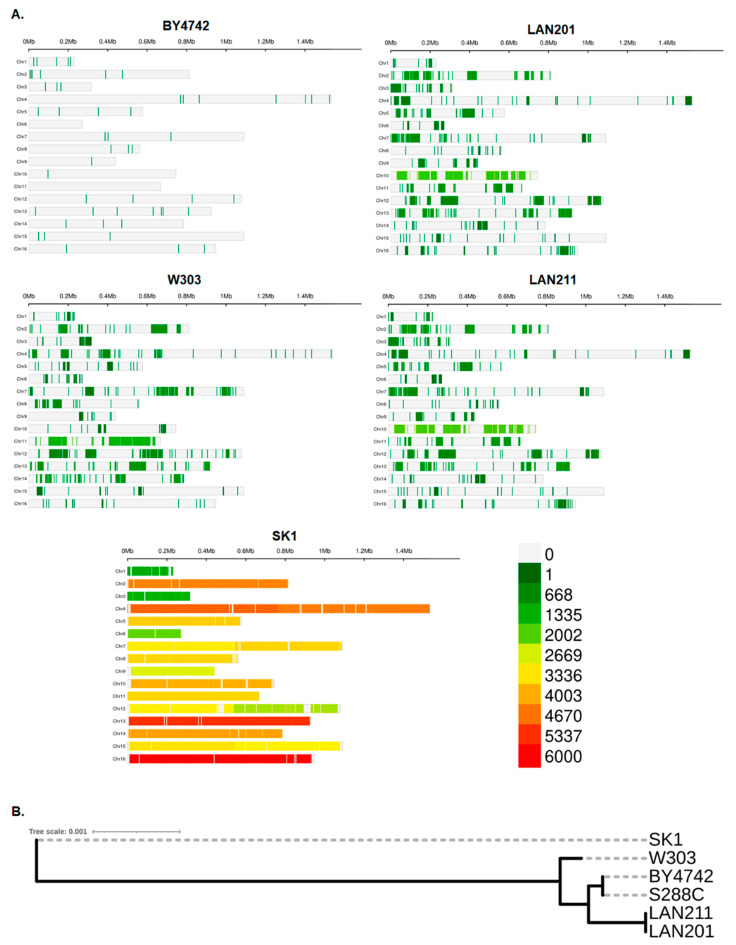
Genome-wide overview of the strain’s differences. (**A**) View of individual chromosomes where regions with a sequence deviation from S288C are shown in a heatmap based on the number of single nucleotide polymorphisms (SNPs) within 1 Mb window size on the 16 chromosomes (the scale 0-6000 is on the bottom right). Analysis was performed with CMplot (Materials and Methods). (**B**) A simple genealogy tree illustrating the considerable evolutionary distance of SK1 from S288C, constructed by REALPHY v.1.13 as described in the Materials and Methods.

We compared whole genome sequencing data of the five strains (data sources are provided in Materials and Methods, Section 4.1, [22,33]) by the methodology described in Section 4.2 and Section 4.3. The results, expressed as differences from the canonical S288C genome, are summarized in Table 1, left half, and illustrated in Figure 2A. The closest to the S288C is the BY4742 strain, which has only 54 variants leading to amino acid changes, with a few variants per chromosome distributed relatively evenly (there is not a single chromosome without changes, Figure 2). The W303 and LAN series appear to have more differences from S288C, and the three strains are quite similar regarding the number of variants (around 2000). The pattern of mutation distribution in W303 differs from LANs in many cases. The most evident examples are large blocks with around 1000 mutations per Mb at different locations in chromosomes II, VII, and XI (Figure 2). LAN201 and its autodiploid LAN211 are virtually identical, both in pattern of mutation localization and in numbers of mutations. However, the transformation by HO-containing plasmid and diploidization (Figure 1) was not wholly benign and a few changes were accumulated. In the following paragraphs, we will refer to these strains under the umbrella name “LAN”. SK1 has overwhelming eight-fold more differences (Table 1). It is vividly demonstrated by a complete change of color in the panel describing this strain in Figure 2A. Panel Figure 2B illustrates a considerable distance between SK1 and the group of the other strains.

### 2.3. DNA Sequence Variations in Genes of the RRR Panel

Next, we compiled a list of 183 genes relevant to genome stability (arranged alphabetically with a short annotation in Supplementary Table S1). The panel includes genes encoding for DNA polymerases and accessory subunits; other proteins involved in DNA replication, repair, and recombination; nucleotide metabolism; chromatin remodeling; cell cycle; checkpoint; and others. We understand that the list might need to be updated because of so many intertwining processes in the cell, but we believe that we selected most of the important genes. Approximately one-third of the genes in our panel are essential for vegetative growth. Only five genes do not harbor non-synonymic or other significant changes: *NTG1*, *RFA1*, *RIM1*, *UBC13*, and *HAM1.* All other genes possess non-synonymous changes that are predicted (by methods described in Section 4.2) to exert a moderate to high impact on protein function, although they are primarily found in the genome of SK1 (Table 1, right half). The analysis of alterations in the gene of the panel revealed only 3 changes in BY4742, around 100 changes in W303-LANs, and more than 600 changes in SK1, averaging around 4 per gene. The information on the position and types of changes for RRR genes are summarized in Supplementary Table S2 and for all genes in Supplementary Table S3. Variations happen both in essential and non-essential genes. 

### 2.4. DNA Sequence Variations in DNA Polymerase-Related Genes

**Figure 3:**
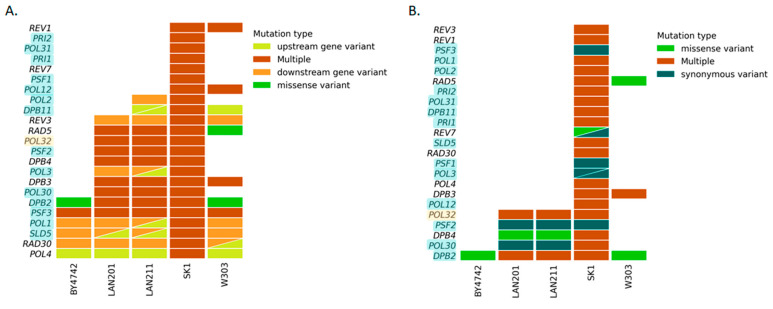
**DNA polymerases and accessory protein-encoding genes: differences from S288C.** Essential genes are highlighted in light blue. One gene, *POL32*, is highlighted yellow because yeast with the deletion of the gene is viable at 30 °C but non-viable at 13 °C (cold-sensitivity) [54]. Deleting the *POL32* ortholog in fission yeast *S. pombe* (*cdc27*) is also lethal [55]. Triangles indicate two variants of the corresponding types (color-coded) in the respective strain/gene combination. Multiple changes include upstream and downstream alterations (5′- and 3′-UTRs), synonymous changes, and missense mutations. (**A**) SNPs and indels: all changes, including promoter, transcription start, and termination zone sequences. (**B**) SNPs and indels in coding regions.

DNA replication is a major characteristic of life. The variation in the genes for the apparatus of replication determines the properties of organisms. Due to our long-term interest in replication [47,48], we focused on polymorphisms of the DNA polymerase-related genes in the five strains (Figure 3). Non-synonymic changes and multiple mutations were prevalent in all the selected genes in SK1 irrespective of essentiality. On the contrary, BY4742 possessed almost no differences from S288C; the only affected essential genes were *PSF3* (although multiple changes do not lead to amino acid changes in the protein, Figure 3B, Supplementary Table S2), a component of the GINS complex necessary for initiation of replication [49] and *DPB2*, a second subunit of pol ε [50]. Both genes are related to the leading DNA strand replication. LANs and W303 possess an intermediate number of variations. Table 2 lists the detected amino acid changes. Not all of the multiple changes listed in Figure 3 cause amino acid substitutions, but they can alter a gene’s transcription or mRNA stability (see details of these changes in Supplementary Table S2). These changes are inherited from CG379 because all the changes in the LAN strains listed in Table 2 are also present in another direct descendant of CG379, strain ySR128 (https://www.ncbi.nlm.nih.gov/nuccore/?term=ySR128 [ncbi.nlm.nih.gov] 29 March 2023) that is extensively used for studies of mutagenic effects of APOBEC deaminases and other agents preferentially damaging ssDNA [51,52,53]. W303 possessed the same amino acid change in Dpb2 as BY4742 and, in addition, a change in Rad5. This finding serves as an internal control for the accuracy of our analysis because the change of Rad5 in this strain was one of the examples of how yeast polymorphisms can affect parameters of genetic processes (Introduction, [7]). No amino acid changes were found in Dpb3, Psf1-3, Pol3, and Pol30, the proteins responsible for bulk replication of the yeast genome. However, another essential component of pol ε, Dpb2, varied in all strains. Conversely, a conditional non-essential Pol32 was stable in BY4742 and W303 but harbored several substitutions in LAN and SK1. Some of these substitutions were shared, alluding to common ancestors.

### 2.5. Genetic Specifics of LAN Strains

**Figure 4:**
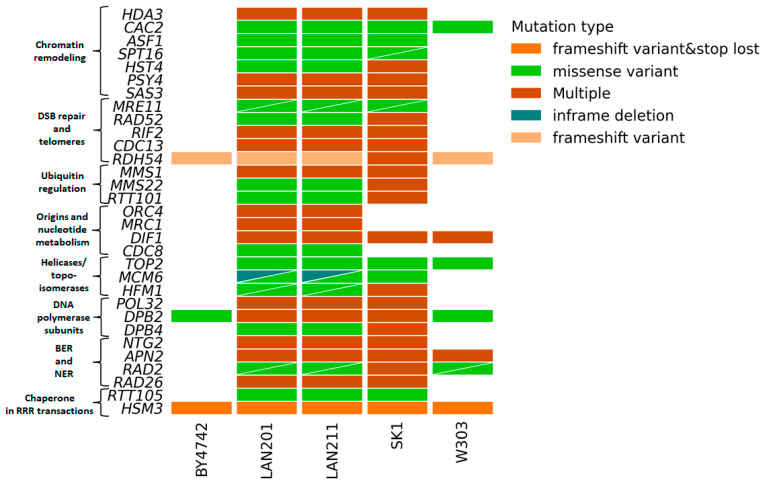
Variations predicted to alter gene function in 183-gene RRR panel in LAN strain series. The analysis was performed as in Figure 3. Triangles indicate two variants of the corresponding types (color-coded) in the respective strain/gene combination.

We were particularly interested in the status of RRR genes in LAN strains because of the extensive work performed by us and others with these strains or their close relatives (Introduction). Figure 4 illustrates the main differences between these strains from S288C and others (details of the analysis results are in Supplementary Table S2). The figure shows what genes are changed in the LAN strains and whether the same genes are polymorphic in other strains. Only a few genes that are variable in LANs are also variable in BY4742 and W303. In contrast, most genes that are variable in LANs are also variable in SK1. The ubiquitous variants across the panel are in the two non-essential genes: *RDH54*, involved in recombination, and *HSM3*, a chaperone involved in DNA mismatch repair, and in one essential gene, *DPB2* discussed in Section 2.4. LAN strains carry variants in all genome stability pathways; the most prominent are in genes participating in chromatin remodeling, DSB repair, DNA polymerases, and cell cycle control. The significance of these variations has to be determined, but the results of our study might help with interpreting and comparing the results of studies performed on different strain backgrounds.

## 3. Discussion

The genetic background of yeast strains plays an insidious or treacherous role in the analysis of genome instability. In addition to numerous known examples of how a single nucleotide change in the genome completely changes the phenotype of the gene under study (some of them are listed in the Introduction), we have our own story. Our present examination of genomes was instigated in part by our recent work on genome-wide analysis of yeast clones that evolved under stress imposed by a deletion of the N-terminal, catalytic active containing half of Pol2, which was performed in LAN201 and E134 strain derivatives [57]. The deletion created in haploid strains confers a severe growth defect, but miserably growing cells yield healthy colonies with time. NGS analysis of the DNA of these fast growers revealed many different genomic variants and recurrent mutations in the essential *CDC28* gene encoding for a catalytic subunit of cyclin-dependent kinase (CDK), a master regulator of the cell cycle. The genetic analysis confirmed that single nucleotide changes in several sites of the gene acquired by the healthy growers cause rescue of the slow growth phenotype (changes L62F, L84V, L86H, and I236N). These changes are located on the surfaces of Cdc28 interacting with cyclins and a Cks1 subunit of CDK. Thus, combining deleterious and benign mutations in two essential genes might provide growth advantages. We did not see any significant changes in the *CDC28* gene in the examined strains. However, *CKS1* has several mutations leading to non-synonymic amino acid changes that are predicted to have a moderate impact on protein function in the SK1 strain (Supplementary Table S2). Thus, the effect of these mutations may be similar to the effect of *CDC28* alleles because they affect the same CDK complex. 

Our work revealed a wide diversity of yeast strains commonly used to analyze genome stability. The genetic consequences of only a few variants have been examined. Many more studies are needed to be performed using structural modeling and genetic and biochemical approaches. Our study will serve as a guidebook for such endeavors and help to better interpret past and future genetic findings.

## 4. Materials and Methods

### 4.1. Sources of Raw Data

The NCBI sources and references to genomic data are in Table 3.

### 4.2. Data Processing

The raw reads of the five yeast strains were filtered by AfterQC (v0.9.7) [58], and adapters, primers sequences, and low-quality nucleotides (*Q* < 20) were removed. Reads shorter than 35 nucleotides after trimming were discarded. The raw and filtered sequences were explored with FastQC to calculate and visualize sequence quality metrics [59]. The resulting filtered reads were then aligned to the S288c reference (R64-3-1) genome using bwa-mem (v0.7.17) with default parameters [60]. Samtools (v.1.17) [61] and picard (v2.27.5) were used to index the reference and create a dictionary. The mapped reads were sorted with samtools (v1.17) [61]. Duplicated reads were marked with Picard MarkDuplicates [62]. Alignment statistics were assessed using picard and samtools. 

The alignment results were used to identify SNPs and indels within each genome by GATK HaplotypeCaller (v4.3.0.0) with sample_ploidy set to 1 or 2 depending on yeast strain ploidy [63]. GATK VariantFiltration was used to filter the vcf files with the following parameters: “QD < 2.0” –filter-name “QD2” -filter “QUAL < 30.0” –filter-name “QUAL30” -filter SOR > 3.0” –filter-name “SOR3” -filter “FS > 60.0” –filter-name “FS60” -filter “MQ < 40.0” –filter-name “MQ40” for snps filtration and QD < 2.0” –filter-name “QD2” -filter “QUAL < 30.0” –filter-name “QUAL30” -filter “FS > 200.0” –filter-name “FS200” for indel filtration. The variant call set was filtered by excluding multiallelic variants, thresholds DP < 10 for read depth, and allele depth AD < 5 for the alternative allele by bcftools (v1.17) [61]. Variants were annotated using snpEFF (v5.1) [64] and Ensembl Variant Effect Predictor (VEP) (v109) that uses multiple criteria to connect sequence variants to the predicted effect on gene function [65].

### 4.3. Oher Methods

SNP-based phylogenetic trees from whole genome sequence data were reconstructed using REALPHY by aligning the reads to S288C reference sequences with default parameters (v.1.13) [66]. The Interactive Tree Of Life (https://itol.embl.de) [67] was used to display of phylogenetic tree. 

The number of SNPs within a sliding window of a 1 Mb size and SNP density plot was carried out with CMplot [61]. Genomic information was visualized with CoMut [68].

The summary of all genomic differences (not only RRR-panel) in our five strains is in Supplementary Table S3. The results for genes that we might have inadvertently not covered above can be found in this Table.

## Figures and Tables

**Table 1:** Genomic view of polymorphism of yeast strains: differences from S288C.

	Whole Genome, SNPs, and Indels			The 183-Gene Panel of RRR Genes		
Sample Name	Total Sequence Variants	Variants with Changes in ORFs	Variants Leading to Amino Acid Changes	Number of Sequence Variants	Variants with Changes in ORFs	Variants Leading to Amino Acid Changes
**BY4742**	147	71	54	31	3	3
**W303**	9412	5414	2115	1787	220	76
**LAN201**	10,492	6149	2366	2265	266	104
**LAN211**	10,999	6246	2425	2286	266	104
**SK1**	77,065	45,093	16,700	13,118	1879	682

**Table 2:** Non-synonymous amino acid differences of the five strains from S288C in DNA polymerases and accessory proteins.

	Strain		BY4742	W303 *	LAN211/LAN201	SK1
Protein		Role				
Pol1 ^§^		Catalytic subunit of pol α, B-family				T21A; E165D; D253G; L326S; T772S; R1021K; G1062E
Pol12 ^§^		Subunit of pol α				L114I; E202K; R513K; I542M; -A in the first ATG codon
Pri1 ^§^		Catalytic subunit of primase				T144M
Pri2 ^§^		Subunit of primase				I272M; K513E
Pol2 ^§^		Catalytic subunit of pol ε, B-family				N39T; S1382A; I1671V; V1936F; Y2110H
Dpb2 ^§^		Second subunit of pol ε	F458Y	F458Y	F458Y; K521R; V565F; E584Q; T644I	A68V; I105T; G206S; T257A; F458Y; S574A;
Dpb3		Third subunit of pol ε	^¶^			
Dpb4		Fourth subunit of pol ε			V150A	G133D; V150A; +I156
Psf1 ^§^		Subunit of GINS	^¶^			
Psf2 ^§^		Subunit of GINS	^¶^			
Psf3 ^§^		Subunit of GINS	^¶^			
Sld5 ^§^		Subunit of GINS				T22I; K23N; M93I; T185A; D220G
Dpb11		Accessory protein of pol ε				Q72K; Q295H; Q296R; C515Y; Y603C R656K
Pol3 ^§^		Catalytic subunit of pol δ, B-family	^¶^			
Pol31 ^§^		Subunit of pol δ and pol ζ				C43Y
Pol32 ^1^		Subunit of pol δ and pol ζ			A5T; Y7S; T223A; G228E; S274P; R302T	A5T; Y7S; G228E
Pol30 ^§^		Sliding clamp, PCNA	^¶^			
Rev3		Catalytic subunit of pol ζ, B-family				E322D; E327K; N405D; F420L; C426W; V623A; H638D; T1383; C1414Y; Q1440E
Rev7		Second subunit of pol ζ				K90R
Pol4		Pol λ-like pol of X-family				V49I; G171E; T334A; M462K; missing codon for E478; A582G
Rev1		TLS pol of Y-family				H141Q; V236I; G280R; H345N; R488K; K781M; I825V; A942T
Rad30		TLS pol of Y-family				M168I; E344G; Y555C; K616R
Rad5		Protein necessary for TLS and repair		G535R		-G resulting in a frameshift at D45: V64L; I164T; R306C; K363E; V385A; V388A; L492M; E564G; T635N; R898S; V973A

* W303 name is originally a diploid generated by the HO gene transformed W301-18A that gave haploid segregants 1A and 1B possessing different mating types [36,56], but now the name W303 is used for a MATα haploid. ^§^ Essential protein. ^¶^ No differences on the amino acid level found. ^1^—viable at 30 °C but non-viable at 13 °C as described in the legend to Figure 3.

**Table 3:** Yeast genomics data source.

Strain	Sequence Read Archive ID	Ploidy	Reference
LAN201	SRR1919946	haploid	[22]
LAN211	SRR534843	diploid	[22]
BY4742	SRR1569895	haploid	[33]
W303	SRR1569900	haploid	[33]
SK1	SRR4453413	diploid	[33]

## Data Availability

Non-applicable.

## Supplementary Materials

The following supporting information can be downloaded at: https://www.mdpi.com/article/10.3390/ijms24097795/s1.
